# A set of simple methods for detection and extraction of laminarinase

**DOI:** 10.1038/s41598-021-81807-2

**Published:** 2021-01-28

**Authors:** Ananthamurthy Koteshwara, Nancy V. Philip, Jesil Mathew Aranjani, Raghu Chandrashekhar Hariharapura, Subrahmanyam Volety Mallikarjuna

**Affiliations:** Department of Pharmaceutical Biotechnology, Manipal College of Pharmaceutical Sciences, Manipal Academy of Higher Education, Udupi, Karnataka 576104 India

**Keywords:** Protein purification, Microbiology techniques

## Abstract

A carefully designed ammonium sulfate precipitation will simplify extraction of proteins and is considered to be a gold standard among various precipitation methods. Therefore, optimization of ammonium sulfate precipitation can be an important functional step in protein purification. The presence of high amounts of ammonium sulphate precludes direct detection of many enzymatically active proteins including reducing sugar assays (e.g. Nelson-Somogyi, Reissig and 3,5-dinitrosalicylic acid methods) for assessing carbohydrases (e.g. laminarinase (β (1–3)-glucanohydrolase), cellulases and chitinases). In this study, a simple method was developed using laminarin infused agarose plate for the direct analysis of the ammonium sulphate precipitates from *Streptomyces rimosus* AFM-1. The developed method is simple and convenient that can give accurate results even in presence of ammonium sulfate in the crude precipitates. Laminarin is a translucent substrate requiring the use of a stain to visualize the zones of hydrolysis in a plate assay. A very low-cost and locally available fluorescent optical fabric brightener Tinopal CBS-X has been used as a stain to detect the zones of hydrolysis. We also report simple methods to prepare colloidal chitin and cell free supernatant in this manuscript.

## Introduction

β-glucans are non-starch polysaccharides which serve a nutritional as well as structural role in algae, plants and fungi. β-glucanases are the enzymes which act upon β-glucans. Laminarin, a type of β-glucan, is the long-term carbon storage polysaccharide found in marine brown macroalgae and consists of β-(1–3) linked glucose molecules interspersed with β-(1–6) intrachain linkages, and 6-O-branching^1^. Laminarin is structurally and chemically similar to the β-glucans found in the fungal cell wall and is used to immunize plants against fungal pathogens^2,3^. Consequently, laminarin is often used as a substrate to evaluate the fungal cell wall-active enzymes^4^.

Glucanases are important in biological pest control, bioremediation, food industry, production of fungal spheroplast and to produce ethanol^5–9^. Further, β-glucanases, especially the ones that hydrolyze laminarin (Laminarinase; EC 3.2.1.6) have been shown to be an integral part of antifungal defense and offense mechanisms of plants and microbes^10–13^. Additionally, many microbial biocontrol agents are laminarinase producers^14^. Therefore, detection and measurement of laminarinase activity is an important activity for research and commercial purposes.

Ammonium sulfate (AS), a member of the Hofmeister series, continues to be the gold standard among various protein precipitation methods. Except for a few exceptions, AS precipitation is the mildest protein precipitation methods available and does not cause large scale denaturation/loss of activity of proteins^15^. Also, the presence of AS has a stabilizing effect on the proteins and is germicidal. Thus, AS precipitation is often used as a preliminary step in majority of protein purification schemes. This step concentrates the target protein, thereby reducing the sample volume by several folds^16^.

Historically, AS had been the reagent of choice for the precipitation and extraction of laminarinase^8,17–22^. Consequently, a large bulk of literature describe AS precipitation as the preliminary step in protein purification. In the recent times, there is a predictable shift towards cloning of laminarinase gene in recombinant hosts^1,22–25^. Based on a survey of the literature, the strategies for the extraction and purification of laminarinase can broadly be classified into the following categories:

(i) AS precipitation of the supernatant followed by chromatographic methods of purification (gel filtration, ion exchange, affinity and hydrophobic interaction)^8,17–22^.
(ii) Concentration of the supernatant using membrane-based concentrators (e.g. tangential flow filtration device and stirred cell concentrators) followed by chromatographic methods of purification^26–31^.
(iii) Recombinant protein expression and purification by chromatographic methods^1,22–25^.

All the three strategies have their own advantages and disadvantages, but strategies (ii) and (iii) stand out for their substantial weaknesses. For example, in strategy (ii), a substantial investment must be made for acquiring and operating the tangential flow filter and stirred cells with ultrafiltration membrane. The devices are expensive and, also require repeated purchase of membrane material and tangential flow capsule (TFC). Similarly, substantial time, skilled manpower and investment are required for strategy (iii). Despite the drawbacks, recombinant expression of proteins is an unavoidable and most preferred step in protein structural studies by X-ray crystallography or protein NMR spectroscopy^1,32^.

Being a non-specific low-resolution method, AS precipitation results in the co-precipitation of contaminating proteins along with the target protein^33,34^. A certain degree of selectivity could be achieved with AS precipitation if the precipitation profile of the target protein at various salt concentrations is known^16^. Although, enzymes can easily be analyzed using plate assays, direct evaluation of enzyme precipitates containing AS using a plate assay was not attempted until now.

AS interferes with most of the biochemical assays like total reducing sugar assays, colorimetric protein estimation assays and SDS-PAGE. Dialysis step is thus an unavoidable hindrance after AS precipitation^16^. The AS precipitation profile of a new/novel protein is usually unknown. Hence, optimization of the AS concentration would involve a large number of samples that need to be dialyzed^35^. This is an impediment, especially for the untrained workers.

In this study, it is demonstrated that the laminarin plate assay is not perturbed by the presence of AS in the non-dialyzed samples. This was achieved by comparing the plate assay results with that of the reducing sugar assay (3,5-Dinitrosalicylic acid (DNS) assay) of the dialyzed samples. An easily available optical fabric brightener (OFB) Tinopal CBS-X (Disodium 4,4′-bis (2-sulfostyryl) biphenyl) has been used to stain the laminarin plate. Congo red (CR) staining, which has been the cornerstone of polysaccharide-based plate assays, is compared with the OFB staining^36^.

To the best of our knowledge, this is the first report for the use of OFB for staining laminarin infused agarose plate using the laminarinase enzyme from an actinomycete (*Streptomyces rimosus* AFM-1).

## Materials and methods

### Materials

Ammonium sulphate, 3,5-dinitrosalicylic acid (DNS), powdered shrimp chitin, yeast extract powder, malt extract powder and other ingredients used in the preparation of colloidal chitin minimal salts (CCMS) broth were purchased from HiMedia laboratories Pvt. Ltd., Mumbai, India. Laminarin derived from *Laminaria digitata* and 12 kDa cutoff dialysis tubing were procured from Sigma-Aldrich chemicals Pvt. Ltd., Bengaluru, India. Concentrated hydrochloric acid was procured from Merck life science Pvt. Ltd., Mumbai, India. Carboxymethyl cellulose (CMC) was procured from Sisco Research Laboratories Pvt. Ltd., Mumbai, India. An actinomycete strain AFM-1 isolated from marine sediment sample from Udupi district, Karnataka, India was used in this study. AFM-1 was identified as *Streptomyces rimosus* based on phenotypic and 16SrDNA sequencing by National Chemical Laboratory (NCL), Pune, India (GenBank accession No. MT604984). Tinopal CBS-X (generic) and 280 g per square meter (GSM) 65/35 polyester-cotton blend laboratory coat clothing material (LCCM) were procured from the local market. Erma ESM11 stage micrometer and eye-piece graticule were procured from Erma Inc., Tokyo, Japan.

### Methods

#### Determination of average Feret’s pore diameter of LCCM

Stage micrometer and an eyepiece graticule were used to calibrate a compound microscope at 40X magnification. The stage micrometer was replaced with the LCCM and pictures were taken. ImageJ software (National Institutes of Health (NIH), USA) was used to analyze the images^37^. The picture with stage micrometer and eyepiece graticule aligned with each other was used to set the scale (“known distance” function with μm as the unit) in ImageJ. Oval or freehand selection tools were used to manually select the pores in the LCCM pictures. The Feret’s diameters and other dimensional parameters were calculated using the “measure” function of ImageJ^38^.

Three different pictures of LCCM and 97 individual pores were analyzed in a similar manner. The results were compiled and the final average Feret’s diameter and standard deviation were calculated.

#### Preparation of colloidal chitin (CC)

**Figure 1:**
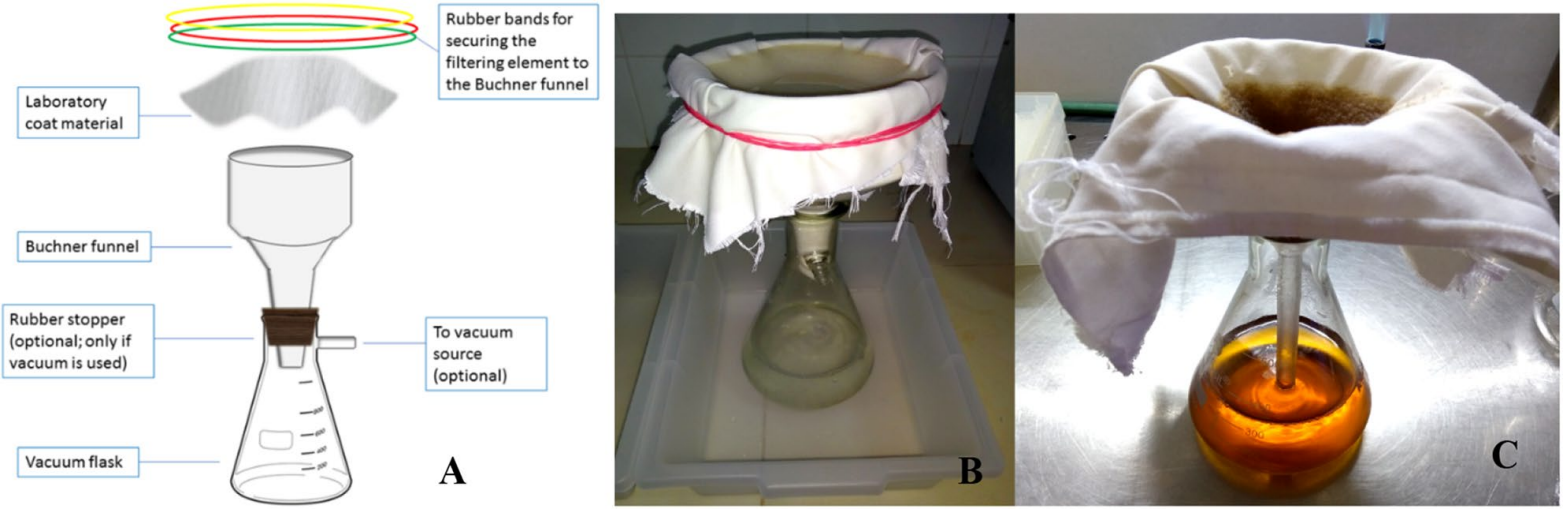
Apparatus used for the preparation of colloidal chitin (CC) and cell free supernatant (CFS). (**A**) The components of the lab coat clothing material (LCCM) based filtration apparatus. (**B**) Filtration setup used for the preparation of CC. Note: A tray was used to collect the liquid dripping from the LCCM due to wicking. (**C**) Buchner funnel was replaced with a glass funnel for the preparation of CFS.

To circumvent the requirement for large volume centrifugation, a filtration apparatus assembled from locally available materials was used for the preparation of CC and cell free supernatant (CFS). LCCM of 280 GSM was used as the filter element. 10 g of powdered shrimp chitin was treated with 100 mL of concentrated 37% w/w hydrochloric acid in a glass beaker kept in a tray filled with cold water. The resultant highly viscous mixture was manually stirred every 5 min with a glass rod for 30 min. The slurry was slowly added to two liters of chilled distilled water with concomitant mixing for 15 min^39^. This thick suspension was passed through the filtering apparatus with LCCM as the filtering element (Fig. 1A,B). The filtration apparatus was setup as depicted in Fig. 1A. The filtration element of the apparatus comprised of a piece of LCCM of sufficient dimensions. A porcelain Buchner funnel (5.6″ diameter) with fixed perforated plate was used for the preparation of CC. The LCCM was fixed to the Buchner funnel using rubber bands.

After the water was drained, the CC paste was collected from the LCCM and resuspended in distilled water and filtered as before. This process was repeated till the pH of CC was near neutral. Then the paste was sterilized by autoclaving and stored at 4 °C until further use.

#### Production of laminarinase

*Streptomyces rimosus* AFM-1 was used to co-produce chitinase and laminarinase enzymes by submerged fermentation. Spore suspensions were prepared from 15 days old yeast extract malt extract glucose (YEME) agar slants. The sporulated slats were gently washed with 5 mL of sterile distilled water containing 0.01% Tween 20 and the suspension was filtered through a sterile custom-made glass wool filter to remove large particles. Custom-made glass wool filter consisted of a 15 mL polypropylene tube filled with glass wool and with a small hole in the bottom of the tube to collect the filtrate. Final spore count was adjusted to 1 × 10^6^ spore mL^−1^ using a Neubauer chamber under light microscope. The inoculum size was 1% and fermentation was carried out in 500 mL Erlenmeyer flasks containing 150 mL of CCMS broth medium with the following composition (L^−1^) 2.803 g K_2_HPO_4_, 1.893 g KH_2_PO_4_, 1.5 g NaCl, 3 g yeast extract, 0.5 g MgSO_4_.5H_2_O, 0.01 g FeSO_4_.7H_2_O, 0.001 g ZnSO_4_, 25 g moist CC and pH 7.0. The fermentation was carried out for 5 days at 28 °C in a shaker incubator set at 150 RPM. CFS was prepared by separating the cell mass using the LCCM based filtering apparatus as described earlier. A glass funnel was used for the preparation of CFS (Fig. 1C). Filtration apparatus and LCCM used for the preparation of CFS were sterilized in an autoclave for 20 min before use.

#### Ammonium sulfate precipitation and dialysis

All steps were performed at 4 °C unless otherwise stated. Fifty milliliters of CFS was used for precipitation with different concentrations of AS. Required quantity of solid AS was slowly added with concomitant mixing using a magnetic stirrer. The AS treated solutions were kept undisturbed for either 20 min (short term incubation; STI) or 3 h (long time incubation; LTI) or 8 h (extended time incubation; XTI). Protein pellet was collected by centrifugation at 4032xg for 30 min. The resultant protein pellets were dissolved in 800 μl of 50 mM potassium phosphate buffer (pH6.5) and either directly used for plate assay (laminarin and carboxy methyl cellulose (CMC)) or dialyzed for the reducing sugar assay. For the dialyzed samples, protein solutions containing AS were dialyzed against 50 mM potassium phosphate buffer (pH6.5) with three successive buffer changes over the period of 12 h using a 12 kDa cutoff membrane tubing. The dialyzed solutions were carefully collected, and their volumes measured.

#### Estimation of laminarinase activity by reducing sugar assay

The laminarinase activity was estimated using a modified protocol of Zhu et al*.,* 2008 with 0.3% laminarin in 50 mM potassium phosphate buffer (pH 6.5) as the substrate^40^. The activity was analyzed after 37 °C for 180 min incubation of reaction mixture containing 0.25 mL of substrate and 0.25 mL of enzyme solution. Formation of reducing sugars was quantified by the 3,5-dinitrosalicylic acid (DNS) method by measuring the color change at 540 nm using a microplate reader^41^. Non incubated reaction mixture served as the blank. Assays were carried out in triplicates. One unit of laminarinase activity is defined as the amount of enzyme that catalyzes the liberation of reducing sugar equivalent to 1 µM d-glucose per minute under standard assay conditions. One unit of chitinase activity is defined as the amount of enzyme that catalyzes the liberation of reducing sugar equivalent to 1 µM N-acetyl-d-glucosamine (GlcNAc) per minute under standard assay conditions.

#### Estimation of laminarinase by plate assay

**Figure 2:**
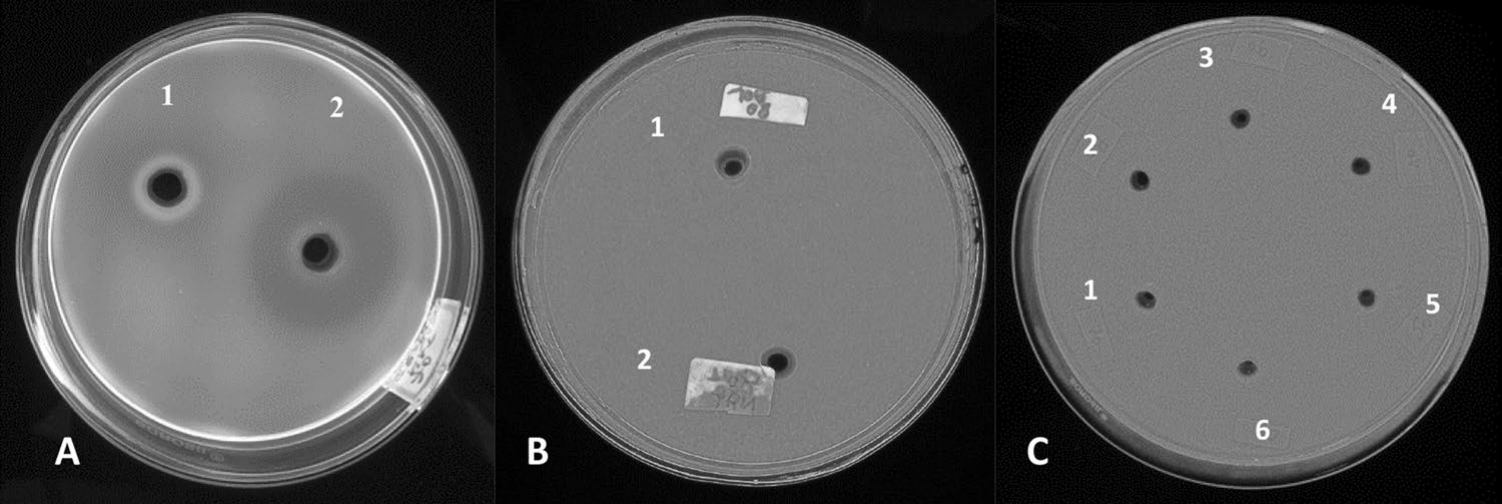
Demonstration of selectivity and reliability of laminarin plate assay. (**A**) 90% saturation ammonium sulfate (AS) precipitate heat treated for 20 min in a boiling water bath (1); 90% saturation AS precipitate non-heat treated (2). (**B**) Heat treated (1) and non-heat treated (2) 90% saturation AS precipitate in a 0.5% carboxymethyl cellulose (CMC) agarose plate. (**C**) Precipitates from various treatment groups (40%-90% AS) tested in a 0.5% CMC agarose plate. 40% (1); 50% (2); 60% (3); 70% (4); 80% (5) and 90% (6). Staining protocol and other parameters for CMC plate assay were same as the laminarin plate assay.

**Figure 3:**
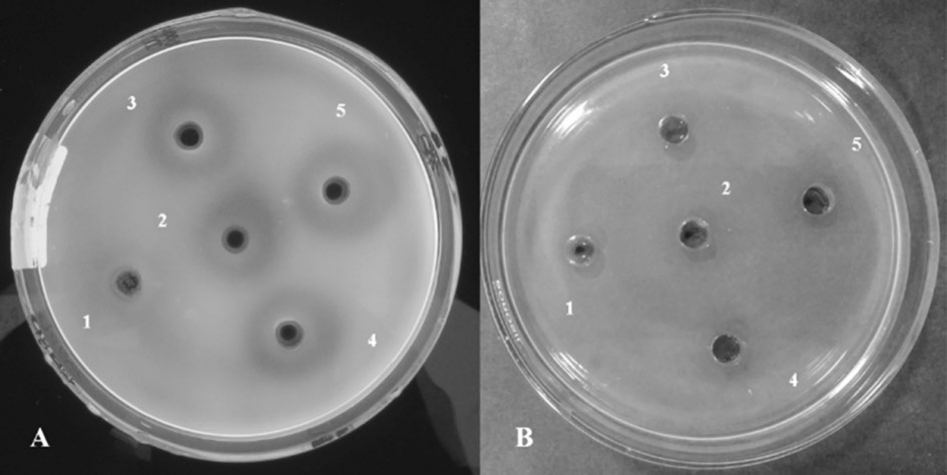
Superior resolution of optical fabric brightener (OFB) vs. congo red (CR) as a stain for the laminarin plate assay. Short Time Incubation (STI) samples (60%-90% AS treatment) were used as the enzyme source. (**A**) Plate stained with OFB and observed under UV light. Boiled sample 90% (1); 60% (2); 70% (3); 80% (4); and 90% (5). (**B**) Plate stained with CR and observed under white light. Boiled sample 90% (1); 60% (2); 70% (3); 80% (4); and 90% (5).

**Figure 4:**
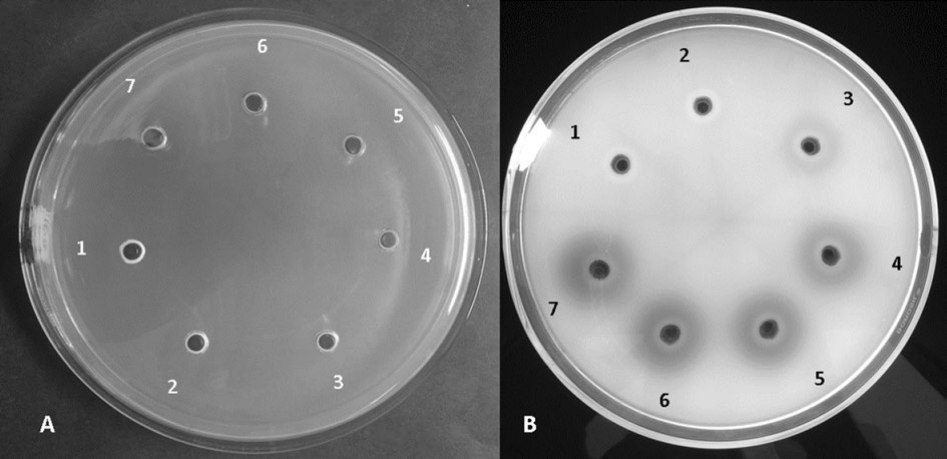
Representative images of laminarinase plate assay for Short Time Incubation (STI) samples (30%-90% AS treatment). (**A**) Laminarinase plate under visible white light. 30% (1); 40% (2); 50% (3); 60% (4); 70% (5); 80% (6) and 90% (7). (**B**) Laminarinase plate under UV light (365 nm). 30% (1); 40% (2); 50% (3); 60% (4); 70% (5); 80% (6) and 90% (7).

Plate assays were carried out in petri dish of either 3.9″ (Figs. 2A,B, 3A,B) or 5.9″ diameter (Figs. 2C, 4A,B). Two plate assay methods were used. For laminarin infused agarose method, plates were prepared as per Michalko et al., 2013 with modifications^42^. The following components (100 mL^−1^) were prepared in 50 mM potassium phosphate buffer (pH 6.5) and used for the plate assay: 0.15 g laminarin and 0.8 g agarose. For CMC plate assay method, laminarin was replaced with 0.5% CMC. The agarose was melted by heating and poured into petri dish to achieve a thickness of approximately 5 mm. After cooling, wells of 6 mm diameter were cut into the agarose gel using a cork borer and 50 μl of protein precipitate solutions from various AS treatment groups were added to the wells. The plates were incubated at 37 °C for 4 h. After the incubation period, the plates were stained for 15 min with freshly prepared 0.15% Tinopal CBS-X in distilled water. The plates were destained with distilled water for 20 min and observed under UV light (365 nm). Assays were carried out in triplicates. CR staining of the laminarin plate was performed with a 0.1% solution of CR for 15 min, destained twice for 15 min with 1 M NaCl and observed under white light^43^.

The zones of hydrolysis were measured using a scale ruler. Pictures of the laminarin and CMC plates (white light and UV) were captured. Heat denatured samples prepared by boiling the AS protein precipitate solution at 100 °C for 20 min was used as control.

## Results

### Preparation of CC and CFS

The 280 GSM 65/35 polyester-cotton blend LCCM was analyzed using ImageJ tool and its average Feret’s diameter was found to be 94.63 ± 7.04 μm^37^.

LCCM had excellent filtering properties, was rugged and could be reused indefinitely for the preparation of CC and CFS. It took approximately 5 min to filter 1liter of CFS and 20 min to filter 2 L of CC slurry. The need for large volume centrifugation facility was avoided by using the LCCM based filtering apparatus. The resultant creamy white colored CC had a soft, pasty consistency with a moisture content of 92–95%. The Buchner funnel was replaced with a glass funnel for the preparation of CFS. A single layer of LCCM was sufficient for the filtration of CC and the fermented broth. The yields of CC and CFS were 93 ± 1.25% and 90 ± 1.47% respectively.

### Production of laminarinase, ammonium sulfate precipitation, dialysis and reducing sugar assay

*Streptomyces rimosus* AFM-1 exhibited pellet growth during submerged fermentation. The productivity of chitinase and laminarinase on the 5^th^ day of fermentation were 11.76 ± 0.86 UmL^−1^ and 13.66 ± 1.24 UmL^−1^ respectively.

**Figure 5:**
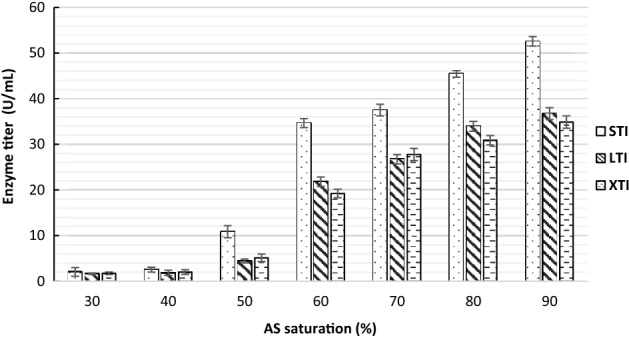
Comparison of laminarinase activity in ammonium sulfate (AS) precipitated and dialyzed samples of *Streptomyces rimosus* AFM-1. Short time incubation (STI) (20 min), long time incubation (LTI) (3 h) and extended time incubation (XTI) (8 h) using DNS assay.

**Figure 6:**
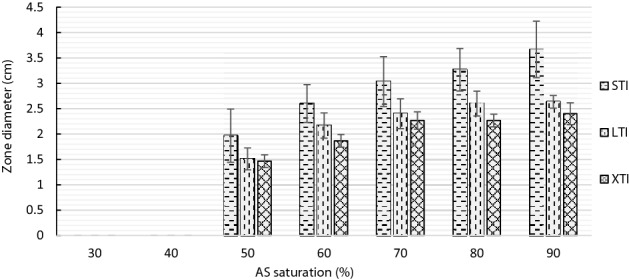
Comparison of zones of hydrolysis of non-dialyzed STI (20 min), LTI (3 h) and XTI (8 h) samples using laminarin plate assay.

The volume of the dialyzed samples increased with increasing AS concentration. The volumes of 90% AS samples were approximately double that of the 40% AS samples. The highest enzyme titers were obtained at 90% AS saturation in STI samples. The enzyme titers across the treatment groups reduced with increasing AS precipitation time and this pattern was similar in both plate assay as well as the reducing sugar assay (Figs. 5, 6).

### Estimation of laminarinase by plate assay

The selectivity and reliability of laminarin plate assay was tested by incubating the AS precipitates in CMC infused plates that were stained with the OFB. No zones of hydrolysis could be detected with either the native or the boiled samples (Fig. 2B,C). Further, boiled enzyme solutions failed to induce zones of hydrolysis in the laminarin plate (Fig. 2A).

The OFB staining protocol used in this study was compared with the CR staining method^43^. Laminarin plates stained with CR had very poor contrast and the bright yellow colored zones of hydrolysis as described in other works of research were missing (Fig. 3B)^42,44^. OFB stain in the laminarin plates was stable for at least 48 h at room temperature. The activity pattern of the various AS treatment groups was clearly represented in the laminarin plate assay (Fig. 4B) and was confirmed by the reducing sugar assay of the dialyzed samples (Fig. 5). The loss of activity with increasing AS precipitation incubation times resulted in reduced zones of hydrolysis (Fig. 6).

## Discussion

Preparation of CC and CFS usually involve large volumes of liquids which need to be centrifuged^45,46^. Large volume centrifugation facilities may not be available in many research laboratories. Filtration is an economical operation compared to large volume centrifugation.

A wide array of filtration elements were used by researchers for the preparation of CC and CFS. Some of the commonly used filtration elements are filter paper, cheesecloth, Miracloth (Calbiochem) and coffee filter paper^47,48^. Filter paper is fragile, non-reusable and has very low filtration rate. When compared to the LCCM, cheese cloth has relatively larger pore size and a small portion of CC is lost during the filtration^48^. Also, cheesecloth does not have the durability of the LCCM. Although perfectly suited for the filtration job, Miracloth costs approximately US$89 for a 4.5 m roll in India. Just like the filter paper, coffee filter paper is fragile and cannot be reused. Moreover, coffee filter paper is not widely available in India^48^.

Under these circumstances, a filtration apparatus made from locally available materials was assembled to circumvent the large volume centrifugation step in the preparation of CC and CFS (Fig. 1). The most important component of this apparatus is the filtering element which is a piece of laboratory coat clothing material (LCCM) of sufficient dimensions to completely cover the funnel. A wide variety of fabric materials are used in the manufacturing of laboratory coat including 100% cotton, cotton-synthetic blends of various proportions and 100% synthetic fabric. Propriety synthetic fibers (e.g. Nomex) are also used for specific purposes. Although, 65/35 polyester-cotton blend was used in this study, LCCM made of 40/60 and 80/20 polyester-cotton blend are also commonly used as they are lightweight, resist wrinkles, and durable^57^.

Buchner funnel was replaced with a glass funnel as it is bulky and consumed more space during sterilization. The use of LCCM for the preparation of CFS is more suitable for fungi and actinomycetes which have pelleted growth in submerged fermentation^49,50^. A single layer of LCCM was found to be sufficient for filtering CC and the fermented broth. Although, not used in this study, a vacuum pump could optionally be used to expedite the process. Some of the advantages of LCCM are as follows:

i) Durability and rugged nature
ii) Very high filtration rate
iii) Reusable
iv) Economical
v) No leakage of CC and microbial cell mass (no bleeding)

The superior functionality of the LCCM as a filter element was abundantly demonstrated not only by the observations listed above, but also a simple wash with soap and water was enough to rejuvenate the LCCM for reuse.

A major disadvantage with the optimization of AS precipitation is that it requires careful handling while dispensing protein solution into and out of the dialysis bag. Any discrepancies will result in either the accidental loss of sample or dilution of the protein solution with buffer. Consequently, the results will be inaccurate. Additionally, performing dialysis for a large number of samples is laborious. Performing a reducing sugar assay after the dialysis step adds to the complexity and time factor of the scheme^16^.

Plate assays can be used to evaluate enzymes, but the use of translucent substrates, such as laminarin, requires the use of a stain to detect the zone of hydrolysis^42,51–53^. Traditionally, CR has been used as a stain to detect the zone of hydrolysis in (1–3) (1–4)-β-d-glucans, O-(hydroxyethyl) cellulose (HEC), and xyloglucan. Surprisingly, CR is a poor stain for CMC and laminarin due to narrow bathochromic shift (< 20 nm)^36,54^. So, a robust and highly selective staining method for laminarin plate assay is required.

An easily available OFB Tinopal CBS-X has been used as a stain in this study. Generic, 99% pure, OFB used in this study costs just US$2 for 100 g. Tinopal CBS-X in the form of a commercial formulation (Ranipal) had been previously used to detect chitinase activity in agarose plate^52^. Ranipal is an admixture of bluing agent and Tinopal CBS-X manufactured by Pidilite Industries Ltd., Mumbai, India. Ranipal is not suitable for staining laminarin plate as the bluing agent reduces the contrast^55^.

A plate assay which is not affected by the presence of AS would simplify the optimization process. Such an idea was the primary motivation for this work. We have demonstrated the reliability and usability of laminarin infused agarose plate assay for the direct quantification of the laminarinase enzyme without the dialysis step. A simple and economical OFB stain was used to visualize the zones of hydrolysis. CR staining is the method of choice for the staining of carbohydrate polymers, but is not recommended for the quantification of laminarin due to narrow bathochromic shift^36^. Consequently, our initial study with CR staining provided poor contrast and inability to properly measure the zones of hydrolysis (Fig. 3B). The OFB used in this study provides better resolution in comparison to CR staining (Fig. 3A). Recently, chromogenic laminarin substrate has also been synthesized^56^. A summary and comparison of various assay methods for laminarinase enzyme is provided in Table 1.

**Table 1:** Comparison of assay methods for laminarinase enzyme.

Assay medium	Assay method	Advantages	Disadvantages
Agarose plate	Laminarin plate assay with optical fabric brightener (OFB) staining	(i) Simple (ii) Economical (iii) Relatively good contrast (iv) Does not require dialysis for ammonium sulfate (AS) treated samples (v) Requires less time (vi) Assay requires commonly available ingredients	(i) Does not directly provide quantitative (U/mL) data (not required for AS optimization)
Agarose plate	Laminarin plate assay with congo red staining	(i) Simple (ii) Economical (iii) Requires less time	(i) Poor contrast due to narrow bathochromic shift (< 20 nm) (ii) Does not directly provide quantitative (U/mL) data (not required for AS optimization)
Liquid	3, 5-dinitrosalicylic acid (DNS) and such other reducing sugar assay methods	(i) Provides quantitative (U/mL) data (ii) Standardized and historically more accepted	(i) Requires dialysis of AS treated samples (ii) Requires a host of reagents. Protocol complexity varies widely (iii) Relatively more tedious and time consuming (iv) Plate assay more suitable for the optimization of AS concentration (v) May provide spurious results due to interference from certain components in the reaction mixture
Liquid and agarose plate	Chromogenic laminarin substrate	(i) Requires less time (ii) Simple colorimetric assay	(i) Chromogenic substrates are relatively more expensive (ii) Can be proprietary (iii) Commercial version of the product not available (iv) Requires chemical synthesis (v) May require dialysis of AS treated samples

The correlation between the zone sizes and enzyme titer was confirmed by reducing sugar assay of the dialyzed samples (Fig. 5). Further, boiled enzyme solution failed to induce zones of hydrolysis in the laminarin plate (Fig. 2A), thus clearly demonstrating its usability. As discussed earlier, Ranipal is not a good choice for the staining of the laminarin plate and is difficult to procure due to limited availability.

In the AS infused CFS, a pattern of progressive reduction in the enzyme activity was seen with the increasing incubation period (i.e. 20 min > 3 hr > 8 hr). This pattern was clearly documented in both plate assay as well as the reducing sugar assay (Figs. 5, Fig. 6). Reduction of the laminarinase activity with increasing AS incubation time indicates possible aggregation or denaturation of the laminarinase^15^. This is an important parameter in deciding the optimal time of precipitation for AS infused samples.

## Conclusion

A set of simple, robust and economical methods for the preparation of CC, CFS and for the direct quantification of laminarinase was developed.

## Supplementary Information

Supplementary Information.
