# Supplementary material for: A set of simple methods for detection and extraction of laminarinase
Source: Sci Rep. 2021 Jan 28;11:2489. doi: 10.1038/s41598-021-81807-2 (PMC7844030; doi:10.1038/s41598-021-81807-2)
Supplement: Supplementary file 1 — Supplementary Information. [file 41598_2021_81807_MOESM1_ESM.docx]

## A set of simple methods for detection and extraction of laminarinase

Ananthamurthy Koteshwara, Nancy V Philip, Jesil Mathew A, Raghu Chandrashekhar H and V.M. Subrahmanyam*

Department of Pharmaceutical Biotechnology, Manipal College of Pharmaceutical Sciences, Manipal Academy of Higher Education, Udupi, Karnataka 576104, India

*Corresponding author: vm.subra@manipal.edu

**Supplementary material**


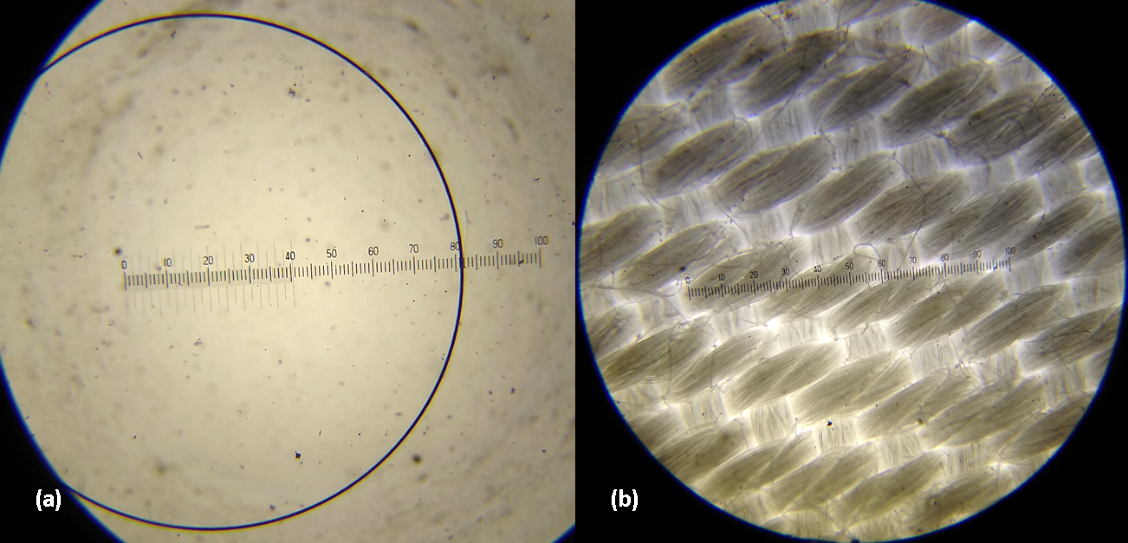


**Fig. 1** ImageJ analysis of laboratory coat clothing material (LCCM). Compound microscope images were capture at 40X magnification. (a) Picture with stage micrometer and eyepiece graticule aligned. (b) Stage micrometer was replaced with LCCM and Feret’s diameters of pores were measured using ImageJ tool

**Fig. 2** Percentage distribution of Feret’s pore diameter in LCCM by ImageJ analysis

| **DNS assay** | | | | | | | | | | | | | | | |
| --- | --- | --- | --- | --- | --- | --- | --- | --- | --- | --- | --- | --- | --- | --- | --- |
| Ammonium sulfate concentration (%) |  | | |  |  |  | | |  |  |  | | |  |  |
|  | **STI (U/mL)** | | | Mean | SD | **LTI (U/mL)** | | | Mean | SD | **XTI (U/mL)** | | | Mean | SD |
| 30 | 1.58 | 1.38 | 3.12 | 2.027 | 0.952 | 1.55 | 1.52 | 1.84 | 1.637 | 0.144 | 1.55 | 1.48 | 2.11 | 1.714 | 0.281 |
| 40 | 1.95 | 3.02 | 2.61 | 2.527 | 0.540 | 1.69 | 2.57 | 1.23 | 1.830 | 0.556 | 1.96 | 2.66 | 1.38 | 2.000 | 0.523 |
| 50 | 10.33 | 12.35 | 9.86 | 10.847 | 1.323 | 3.88 | 4.86 | 4.57 | 4.437 | 0.411 | 5.14 | 6.11 | 4.09 | 5.113 | 0.825 |
| 60 | 33.62 | 35.55 | 34.77 | 34.647 | 0.971 | 22.54 | 22.52 | 20.33 | 21.797 | 1.037 | 20.00 | 17.89 | 19.78 | 19.223 | 0.947 |
| 70 | 36.35 | 38.88 | 37.25 | 37.493 | 1.282 | 27.28 | 25.30 | 27.62 | 26.733 | 1.023 | 29.20 | 28.11 | 26.01 | 27.773 | 1.324 |
| 80 | 45.00 | 46.25 | 44.98 | 45.410 | 0.727 | 33.14 | 33.20 | 35.45 | 33.930 | 1.075 | 32.08 | 31.03 | 29.59 | 30.900 | 1.021 |
| 90 | 51.64 | 53.70 | 52.35 | 52.562 | 1.047 | 35.79 | 35.83 | 38.56 | 36.727 | 1.296 | 35.39 | 36.23 | 33.01 | 34.877 | 1.364 |

**Table 1.** Assay values of 3,5-dinitrosalicylic acid (DNS) test for dialysed ammonium sulfate (AS) treated cell free supernatant (CFS). SD: Standard Deviation

| **Plate assay** | | | | | | | | | | | | | | | |
| --- | --- | --- | --- | --- | --- | --- | --- | --- | --- | --- | --- | --- | --- | --- | --- |
| Ammonium sulfate concentration (%) | **STI (cm)** | | | Mean | SD | **LTI (cm)** | | | Mean | SD | **XTI (cm)** | | | Mean | SD |
| 30 | 0.00 | 0.00 | 0.00 | 0.000 | 0.000 | 0.00 | 0.00 | 0.00 | 0.000 | 0.000 | 0.00 | 0.00 | 0.00 | 0.000 | 0.000 |
| 40 | 0.00 | 0.00 | 0.00 | 0.000 | 0.000 | 0.00 | 0.00 | 0.00 | 0.000 | 0.000 | 0.00 | 0.00 | 0.00 | 0.000 | 0.000 |
| 50 | 1.70 | 1.50 | 2.70 | 1.967 | 0.525 | 1.50 | 1.25 | 1.78 | 1.510 | 0.216 | 1.50 | 1.30 | 1.60 | 1.467 | 0.125 |
| 60 | 2.20 | 2.50 | 3.10 | 2.600 | 0.374 | 2.10 | 1.90 | 2.50 | 2.167 | 0.249 | 2.00 | 1.90 | 1.70 | 1.867 | 0.125 |
| 70 | 2.40 | 3.10 | 3.60 | 3.033 | 0.492 | 2.30 | 2.10 | 2.80 | 2.400 | 0.294 | 2.20 | 2.10 | 2.50 | 2.267 | 0.170 |
| 80 | 2.70 | 3.70 | 3.40 | 3.267 | 0.419 | 2.60 | 2.30 | 2.90 | 2.600 | 0.245 | 2.30 | 2.40 | 2.10 | 2.267 | 0.125 |
| 90 | 2.90 | 3.90 | 4.20 | 3.667 | 0.556 | 2.60 | 2.50 | 2.80 | 2.633 | 0.125 | 2.30 | 2.20 | 2.70 | 2.400 | 0.216 |

**Table 2.** Laminarin infused agarose plate assay values of non-dialysed AS treated CFS. SD: Standard Deviation
